# An Insight into Abiotic Stress and Influx Tolerance Mechanisms in Plants to Cope in Saline Environments

**DOI:** 10.3390/biology11040597

**Published:** 2022-04-14

**Authors:** Zarmina Gul, Zhong-Hua Tang, Muhammad Arif, Zhang Ye

**Affiliations:** 1 College of Chemistry, Chemical Engineering and Resource Utilization, Northeast Forestry University, Harbin 150040, China; yzhang@nefu.edu.cn; 2 Key Laboratory of Eco-Environments in the Three Gorges Reservoir Region (Ministry of Education), Chongqing Key Laboratory of Plant Resource Conservation and Germplasm Innovation, College of Life Sciences, Southwest University, Chongqing 400715, China; muhammadarif@swu.edu.cn

**Keywords:** halophytes, glycophytes, SOS pathway and HKT channels, sensing, ionic homeostasis, osmolytes

## Abstract

**Simple Summary:**

This review focuses on plant growth and development harmed by abiotic stress, primarily salt stress. Salt stress raises the intracellular osmotic pressure, leading to hazardous sodium buildup. Plants react to salt stress signals by regulating ion homeostasis, activating the osmotic stress pathway, modulating plant hormone signaling, and altering cytoskeleton dynamics and cell wall composition. Understanding the processes underlying these physiological and biochemical responses to salt stress could lead to more effective agricultural crop yield measures. In this review, researchers outline recent advances in plant salt stress control. The study of plant salt tolerance processes is essential, both theoretically and practically, to improve agricultural output, produce novel salt-tolerant cultivars, and make full use of saline soil. Based on past research, this paper discusses the adverse effects of salt stress on plants, including photosynthesis suppression, ion homeostasis disturbance, and membrane peroxidation. The authors have also covered the physiological mechanisms of salt tolerance, such as the scavenging of reactive oxygen species and osmotic adjustment. This study further identifies specific salt stress-responsive mechanisms linked to physiological systems. Based on previous studies, this article reviews the current methodologies and techniques for improving plant salt tolerance. Overall, it is hoped that the above-mentioned points will impart helpful background information for future agricultural and crop plant production.

**Abstract:**

Salinity is significant abiotic stress that affects the majority of agricultural, irrigated, and cultivated land. It is an issue of global importance, causing many socio-economic problems. Salt stress mainly occurs due to two factors: (1) soil type and (2) irrigation water. It is a major environmental constraint, limiting crop growth, plant productivity, and agricultural yield. Soil salinity is a major problem that considerably distorts ecological habitats in arid and semi-arid regions. Excess salts in the soil affect plant nutrient uptake and osmotic balance, leading to osmotic and ionic stress. Plant adaptation or tolerance to salinity stress involves complex physiological traits, metabolic pathways, the production of enzymes, compatible solutes, metabolites, and molecular or genetic networks. Different plant species have different salt overly sensitive pathways and high-affinity K^+^ channel transporters that maintain ion homeostasis. However, little progress has been made in developing salt-tolerant crop varieties using different breeding approaches. This review highlights the interlinking of plant morpho-physiological, molecular, biochemical, and genetic approaches to produce salt-tolerant plant species. Most of the research emphasizes the significance of plant growth-promoting rhizobacteria in protecting plants from biotic and abiotic stressors. Plant growth, survival, and yield can be stabilized by utilizing this knowledge using different breeding and agronomical techniques. This information marks existing research areas and future gaps that require more attention to reveal new salt tolerance determinants in plants—in the future, creating genetically modified plants could help increase crop growth and the toleration of saline environments.

## Contents:

▪ *An overview of abiotic stress; effects of salinity stress on crop growth, development, and yield*
▪ *Salinity—a major limiting factor in the ecosystem and an inhibitor of plant growth*
▪ *Alkaline Salinity (high pH)*
▪ *Classifications of plants; Glycophytes (salt-sensitive plants) and halophytes (salt-resistant plants) are two salinities (salt-tolerant plants)*
▪ *Impact of salinity on photosynthesis and stomatal conductance*
▪ *Causes of soil salinity; signal transduction and ionic homeostasis under salt stress (leading to osmotic, ionic, and oxidative stresses)*
▪ *SOS pathway (salt overly sensitive pathway)—sensing salt stress in plants*
▪ *Channels involved in Na^+^ ion regulation—HKT (High-affinity K^+^ channel), NSCC (Non-selective cation channel), AKT1 (Arabidopsis K^+^ Transporter1), NORC (nonselective outward-rectifying conductance), VIC (Voltage-independent channel)*
▪ *Metabolic profiling; osmolyte production in plants under salt stress; proline, glycine betaine, abscisic acid, jasmonates, flavonoids; plants and rhizosphere microbial activities in response to various stress conditions.*
▪ *Conclusions and future perspectives.*

## 1. Content Description

### 1.1. An Overview of Abiotic Stress; Soil Salinization—A Major Environmental Constraint and Plant Growth Inhibitor

**Figure 1:**
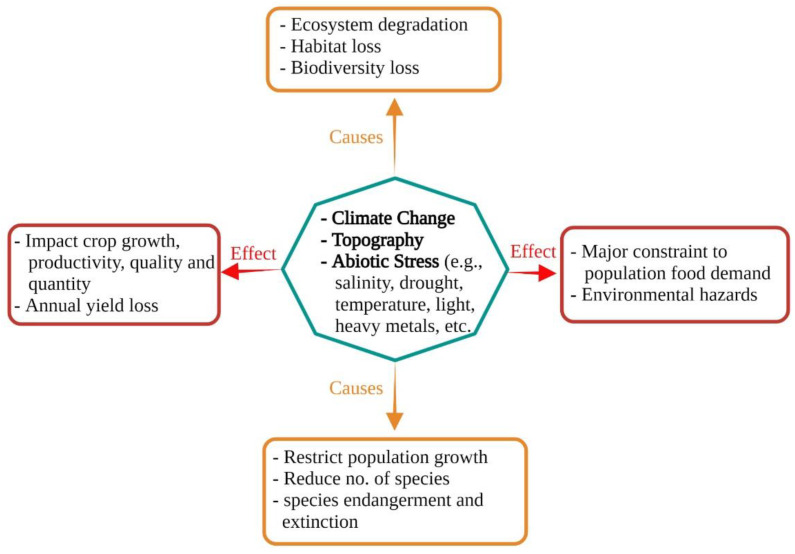
Schematic representation of effects and causes of abiotic stresses on different life forms, ecosystems, and crop yields.

Abiotic stresses like salinity, drought, light and heavy metals, and high temperatures have undesirable consequences for crop productivity, quality, and quantity and induce alarming traits in sustainable agriculture [1]. Salinity is mainly a vital proscribing component, inflicting low yields with inferior quality (Figure 1). There are many factors causing soil salinity. Weather change is considered one of the fundamental contributing elements to soil salinization, including ecosystem degradation, loss of habitat, and desertification [2]. Another essential component contributing to soil salinization is contaminated irrigation water [3], inferring that soil salinization is a very important cause of reductions in the productiveness of the irrigated and rain-fed acreages of the world [4]. Salinity has a negative effect on plant life causing poor growth, mutations, or inhibition of biochemical and physiological mechanisms.

The accumulation of excess soluble salts in the soil is called soil salinization [5]. Arid and semi-arid zones have relatively high [1] soil salinization throughout the year, with a greater evapotranspiration than precipitation rate. Plants have adapted several mechanisms to tolerate excessive amounts of sodium chloride to ensure the availability of other nutrients, such as nitrate, phosphate, potassium, and calcium at low concentrations [6]. Primary soil salinization is also known as natural soil salinization. Because of parent rock intrusion, seawater invasion, or wind salt inclination, it mainly occurs in arid and semi-arid climatic zones. On the contrary, the main source of secondary soil salinization is anthropogenic activities. It is caused by using low-quality irrigation water and the application of agrochemicals. Secondary soil salinization is expanding worldwide, imposing deleterious effects on agricultural productivity [7].

Excess soluble salts in the soil are an adverse environmental factor that affects seed germination, crop growth, and plant productivity [8]. Secondary soil salinization is the main threat to irrigated cultivated farmland. Due to water evaporation, the salt accumulates in the irrigated soil. In some areas, this secondary salt finally leaches out into the groundwater. Salt stress is caused by high sodium (Na^+^) and chloride (Cl^−^) ion concentrations in the soil [9]. In most saline areas, sodium and chloride are the predominant ions. Unlike halophytes, which are salt-tolerant plants, the productivity of glycophytes (salt-sensitive plants) is extremely reduced by salt stress [10]. In plants, several genes control salt tolerance traits through intricate genetic regulatory complexes. Salinity stress further leads to osmotic and ionic stress, consequently affecting plant growth by causing nutritional deficiencies. High salt concentrations consistently lower soil osmotic potential (physiological drought) [11], thus making soil solutions unavailable to plants [12]. Thus, the roots become unable to uptake water from the soil (physiological drought). At the same time, the gradual accumulation of salts in plant tissues over time is associated with ionic stress [8]. Hence, researchers concluded that, in these harsh conditions, plants could only survive through their ability to sense salt stress and then respond appropriately.

Crops that have the lowest electrical conduction (EC) of a saturated soil paste extract (ECe) (a standard soil salinity measurement index used to measure plant salt tolerance) exceeding four decisiemens/meters ultimately exhibit reduced crop yield when grown under high salinity. Salinity is caused by excessive accumulation of calcium, magnesium, potassium, chloride, sodium, and some anions such as carbonates, sulfates, nitrates, bicarbonates. Saline soil exhibits enormous physiochemical properties due to diverse ionic compositions. At the same time, growth is hindered in saline-sodic soils by a high concentration of sodium, elevated alkalinity levels, and higher salt concentrations [13]. On this subject, it is important to discriminate between saline soil and sodic soil. Sodicity is the term given to the amount of sodium apprehended in the soil. Soil sodicity indicates that sodium concentrations are 5% higher than overall cationic concentrations. sodicity stops air and water from moving and causes clay to swell too much when wet, which leads to a poor drainage system [6].

### 1.2. Alkaline Salinity

Alkaline stress, also known as high pH salinity, is one of the plants’ abiotic constraints that coexists with salt stress and results in significant losses in global agricultural productivity. When plants are repeatedly exposed to excessive salinity and pH, the cumulative damage appears to be greater than in a single incidence [14]. Salinity affects around 954 million hectares of land on the planet [15]. Salt stress is caused by neutral salts such as NaCl. Although salt stress is a type of stress, alkaline salt stress is generated by alkaline salts such as NaHCO_3_ and Na_2_CO_3_, which is referred to as alkaline stress, and causes greater damage than neutral salt [16,17]. Although alkaline stress and salt stress have many similarities, such as osmotic stress and ion toxicity, the alkaline situation has three distinct characteristics that should be considered as a separate stress type. High soil pH, Na^+^ toxicity, and water deprivation have a deleterious impact on plant growth and development. High alkaline pH produces oxidative stress in plants by producing reactive oxygen species (ROS) and malondialdehyde (MDA), both of which harm membrane integrity and intracellular components. Plants utilize a variety of enzymatic and non-enzymatic antioxidants to reduce ROS-induced oxidative stress. Superoxide dismutase (SOD), catalase (CAT), peroxidase (POX), and ascorbate peroxidase (APX) are enzyme antioxidants that help to scavenge superoxide radicals and hydrogen peroxide (H_2_O_2_). Furthermore, a few more studies indicated that mild alkaline stress improved sugar beet seedling growth, leaf chlorophyll content, photosynthetic index, and antioxidant activities [18]. Several research organizations around the world have investigated tolerance mechanisms for salt stress responses in diverse crops and model land plants. However, there are only a few studies that look at high salinity combined with alkaline stress [19].

### 1.3. Classification of Plants Based on Salinity: Glycophytes (Salt-Sensitive Plants) and Halophytes (Salt-Tolerant Plants)

**Figure 2:**
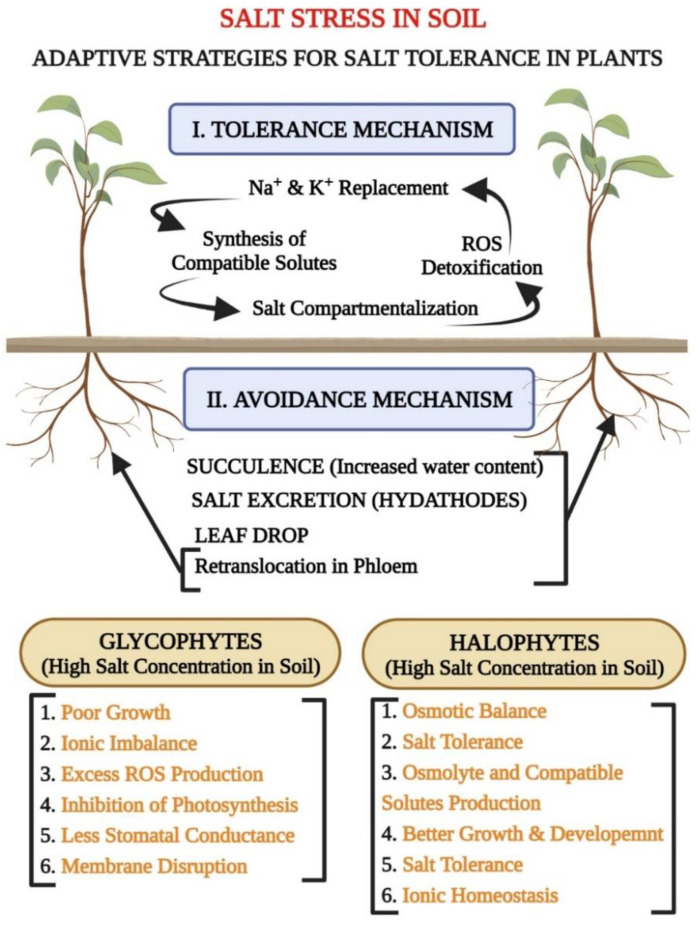
Schematic representation of adaptive strategies for salt tolerance in plants, including tolerance and avoidance mechanisms; the differentiation of plants into glycophytes and halophytes based on their responses to salt stress.

Based on growth in saline soils, plants may be categorized into two groups, glycophytes or halophytes (Figure 2), by determining their capability to survive in excessive salt concentrations [20]. Most crop plant species are glycophytes (mesophytes, hygrophytes, and some xerophytes), which means they can only grow in nonsaline soils and freshwater bodies. Their salt stress tolerance varies by species [10,21]. Halophytes are plants that grow naturally in highly saline soils (salt marshes and mudflats). In comparison to halophytes, salt pressure inhibits glycophyte growth and development. [22]. Globally, salt will affect 20% of all cultivated land and 33% of all irrigated land [1].

This case is further alarming because essential glycophytic crops such as cereals, grains, tomatoes, and potatoes are hypersensitive to saline strain [23]. Glycophytes are plants that are severely affected by saline conditions at both the cellular and whole-plant levels. Under salt stress, glycophytes reveal higher solute accumulation, ionic and osmotic stresses, and negotiate nutritional imbalances, restricting the mass production of those plants. Most plants in the terrestrial ecosystem are glycophytes, including most of the crop flora [1]. Conversely, halophytes modify their biochemical and physiological mechanisms by producing osmolytes and compatible solutes, ionic compartmentalization, biochemical changes, and absorption of beneficial ions. These changes promote seed germination, succulence, and salt exclusion in saline environments [24,25], as well as efficient cytosolic Na^+^ sequestration to vacuoles and salt bladders, retention of higher K^+^ in the cytosol, and effective control of xylem ion loading and unloading, succulence, and salt-secreting glands [26,27].

Remarkably, halophytes also have a greater quantity of mitochondria, indicating that a greater amount of energy is required to endure saline conditions [28]. Halophytes accumulate fewer Na^+^ and Cl-ions in their cytoplasms, allowing their chloroplasts to survive, even when the plant is subjected to severe salinity shockwaves [29]. Halophytes have unique glands for salt excretion that are present on halophytic plant leaves. Before reaching the shoots, these leaves remove the salt from their surfaces. The presence of halophytes is restricted to habitats with plenty of water (e.g., salt marshes). Because of their ability to function effectively, even with seawater irrigation, a few halophytic species, such as quinoa (*Chenopodium quinoa*), which produce extremely nutritious seeds, are thought to be the best for saline agriculture [26]. Quinoa (a halo-phytic crop) contains epidermal bladder cells (EBCs) as an adaptive strategy for salt secretion. The origin of the development of these EBCs is still unclear, even though EBCs are considered like trichome cells in *Arabidopsis Thaliana*. Genetic studies of quinoa EBCs revealed their higher number of ion and sugar transporters and abiotic stress-mediated genes [30]. Now that its genome has been sequenced [31], quinoa is a model halophytic crop species to examine in order to develop further tolerant crops. If these plants thrive in relatively drier habitats, salt hairs (which help in water loss regulation) are re-placed by secretory glands [32].

Another significant adaptation of these plants is hydathodes. These structures help remove immoderate salts with less stomatal conductance and more transpiration water loss [33]. Other than halophytes with innate production of salt-tolerant genes, most crop species only trigger their tolerance phenomenon by revealing a particular stress type. The activation of the tolerance program results in salinity acclimation. It involves altered physiological responses, the redirection of metabolism, the reinforcement of defense and repair, and changes in developmental processes to conform to morphological and anatomical proficiencies. The profound reorganization involves distinguishing cues and modulating pathways. A massive set of studies, particularly with *Arabidopsis thaliana* cultivars and the use of transcript profiling, has assigned critical roles to all plant hormones in the acclimation process, growth, and organ structure markings under salt stress [34]. The above studies and research theories suggest that the salt-tolerance mechanisms of halophytes depend upon their unique morphological, anatomical, and physiological structures and processes, distinguishing them from glycophytes. There is a great need to learn more about these morphological and physiological distinctions in halophytes so that we will be able to produce salt-tolerant glycophytic plants by improving their tendencies to cope with saline conditions. In this way, we can meet the need for food production and agricultural demands.

### 1.4. Impact of Salinity on Photosynthesis and Stomatal Conductance

Elevated salt levels disrupt stomatal conductance and photosynthetic rates in plants. When plants are exposed to salinity, the stomatal aperture decreases to maintain an osmotic balance around the root area [8]. Salt stress reduces stomatal conduction by affecting stomatal openings, their size, and density. Subsequently, the rate of photosynthesis and transpiration drops gradually. Cotton plants subjected to salt stress treatments are observed to have a considerable decline in chlorophyll and distortion of chlorophyll structure, consequently leading to a reduced photosynthetic rate [35]. Low stomatal conductance was noticed in both wild type (Ailsa Craig) and abscisic acid-deficient mutant (notabilis) tomato genotypes, and it was found to be negatively associated with growing xylem ABA in both genotypes [36]. Stomatal conductance is frequently linked to photosynthetic efficiency, which is crucial for increased biomass production and yield [25].

Due to the higher accumulation of sodium (Na^+^) and chloride (Cl^−^) in the chloroplast, photosynthesis is inhibited, especially photophosphorylation and carbon metabolism. At the same time, the photosynthetic electron transport chain (ETC) appears relatively insensitive to salt. The synthesis of adenosine triphosphate (ATP), which provides the energy required for CO_2_ fixation into sugars, relies on photosynthesis. Abiotic stresses disrupt thylakoid membranes, influence the electron transport chain, alter enzymatic activity and protein synthesis, and affect Calvin cycle patterns, all of which affect photosynthetic processes [37]. Most of these abnormalities cause ATP synthesis to be disrupted [38,39], resulting in iron deficiency due to ion degradation and synthesis inhibition [40,41,42]. Cucumber (*Cucumis sativus*) leaves lose relative water content after being exposed to saline solutions [43]. The authors in this study attributed their observations to higher Na^+^ and lower K^+^ materials, which reduce photosynthesis due to antagonistic Na-ion uptake rivalry. When the entire root system is exposed to salt stress, photosynthesis in pepper plants is documented to be lower than when only a portion of the root system is exposed, and stomatal conductance and transpiration are negatively affected by complete or partial salt stress [44]. On the other hand, in saline environments, the rate of photosynthesis is reduced, resulting in the formation of reactive oxygen species (ROS) [45]. Reactive oxygen species (ROS) are essential signaling molecules that monitor multiple organic processes, even though excessive ROS accumulation leads to oxidative stress [46,47]. The reduced photosynthetic rate operates on the activity of enzymatic antioxidants such as catalase (CAT), superoxide dismutase (SOD), and various peroxidases that detoxify ROS [48]. These enzymes work in a coordinated manner to maintain a balance between the formation and removal of ROS.

High salinity ions in the soil solution are characterized by an increase in Na^+^ and Cl-ions, which cause hyperosmotic and infiltration conditions in the soil solution, preventing plants from absorbing water and nutrients [49]. As a result, Na+ removal from the photosynthetic organ is required for proper metabolism and carbon fixation [50]. ROS is a complex networking system that acts as a signaling molecule in plants to control further growth, development, and stress responses. Under these circumstances, plants undergo adjustments in chloroplast pigment compositions, leaf morphology, and biochemical processes to prevent oxidative damage to photosystems. Excessive ROS production inhibits photosynthesis in the leaves of sensitive plants [51]. In addition to reallocating resources for osmotic adjustment, a decrease in photosynthesis reduces available resources, and thus growth, in response to salt. The quantity of CO_2_ available for fixation is reduced by stomatal closure, although increasing the CO_2_ concentration can only partly recover the photosynthetic rate [52]. These findings suggest that there is also an ionic effect or a slight stomatal closure-independent effect of sodium on photosynthesis. During salt stress, the activity of CO_2_-fixing enzymes decreases. This enzyme’s ability to tolerate sodium in vitro varies between species [53]. Despite this, relying on this surveillance for photosynthetic capability has yet to be validated. As a whole, Na^+^ affects photosynthesis by causing problems with chloroplasts and the proton-motive force, as well as CO_2_ fixing enzymes [54].

### 1.5. Signal Transduction and Ionic Homeostasis; Osmotic and Ionic Stress

Maintaining cell ion homeostasis is a vital adaptive attribute of salt-tolerant plants in reaction to excess ions. Under salt stress, K^+^ helps maintain ion homeostasis and control the osmotic balance [55]. An appropriate K^+^/Na^+^ ratio inside the cytoplasm may be attained via reducing cytoplasmic Na^+^ and increasing cytoplasmic K^+^, preventing cellular damage and [56] nutrient deficiency. Mechanisms to lower cytoplasmic Na^+^ include hampering Na^+^ uptake, increasing Na^+^ efflux, and tagging Na^+^ inside the vacuole. It is not fully understood how plants sense salt stress; there is an ongoing debate over whether plants have a sodium sensor or receptor. Salt stress induces osmotic and ionic stress in plants. Consequently, osmotic and ionic stresses raise calcium concentrations in the cytosol. However, in Arabidopsis roots, salt-induced cytosolic calcium levels generally increase in the cortical and endodermal cell stratums, and mannitol-induced elevation in cytosolic calcium levels primarily occurs in the epidermal cell layer [57]. Osmotic stress signaling and abscisic acid (ABA) pathways are also triggered by salt stress. At the same time, salt treatment increases ABA concentrations in plant cells [58,59].

**Figure 3:**
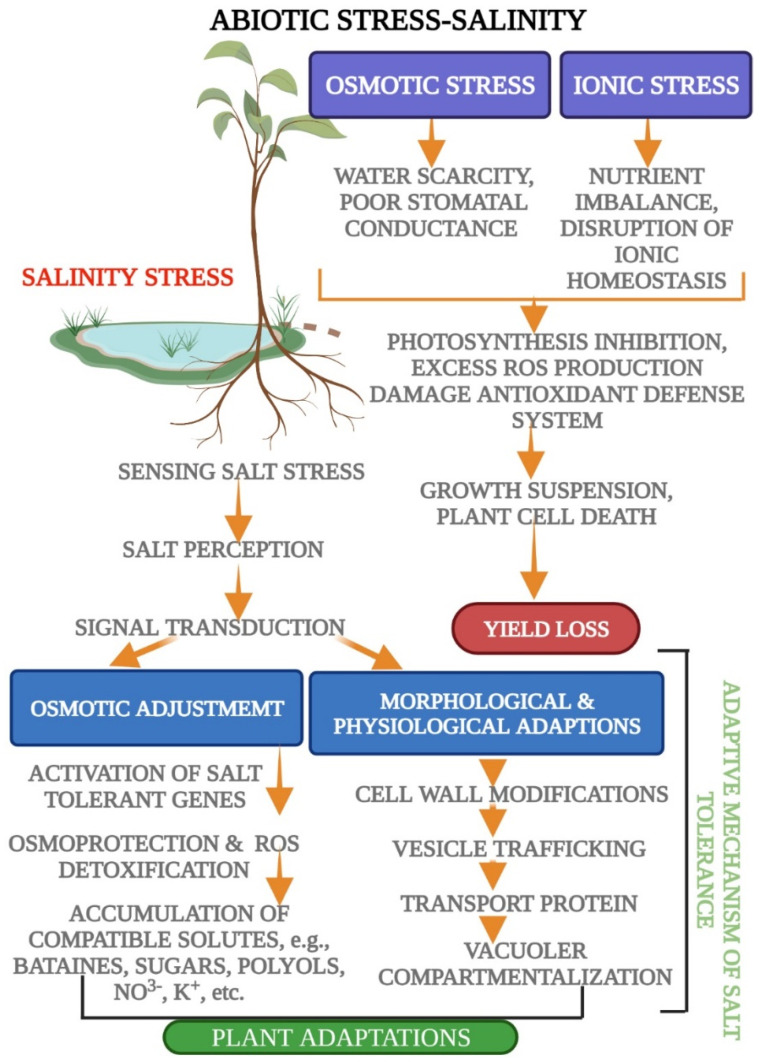
Schematic representation of osmotic and ionic stresses in plants in relation to salt stress. Adaptive mechanisms of plants, including osmotic adjustment and morpho-physiological adaptations, to maintain ionic homeostasis in their cell solutions.

Numerous undesirable effects seem to be due to the high salt gradient. Ionic imbalance is one of the principal domino effects. A high concentration of Na^+^ and Cl-ions, for example, can trigger biochemical processes that are fatal to plant life [60]. Three kinds of signal transduction pathways have mainly been identified under abiotic stress (Figure 3), i.e., the ionic signaling pathway, the osmolyte regulation pathway, and the gene regulation pathway [61]. The ionic stress signaling pathway has been exemplified for signal transduction under salinity strain. Calcium (Ca^+^) occupies an important role in this regard. It induces signal transduction in plant life to adapt to stressful situations [62]. Gene expression, ionic adjustments, enzymatic activities, accumulation of compatible solutes, and other processes are activated by increasing cytosolic calcium levels [63]. Moreover, the exogenous application of calcium regulates K^+^/Na^+^ selectivity and, accordingly, confers salt tolerance by enhancing signal transduction. In salt-stressed situations [64,65], glycinebetaine is suggested to maintain signal transduction and ion homeostasis. These results suggest that sodium and osmotic sensors are involved in perceiving excess salt in different cell types. Arabidopsis roots that are exposed to too much NaCl build up high concentrations of Na^+^ in root tissues in the first two minutes.

On the other hand, Na^+^ efflux from the root tissue also begins [66]. This research highly supports the evidence that excess Na^+^ is instantly detected by plants, which generates downstream sodium-stress responses. In this regard, the salt overly sensitive (SOS) pathway in plants is of great importance in aiding in the removal of excess Na^+^ ions. Within two hours of the exposure of plants to salt stress, the SOS pathway immediately activates. When the SOS1 Na^+^ antiporter (which increases sodium efflux) and the SOS2 kinase (which increases sodium efflux) are activated, they send sodium ions out of the plant cells.

### 1.6. SOS Pathway (Salt Overly Sensitive Pathway)—Sensing Salt Stress in Plants

**Figure 4:**
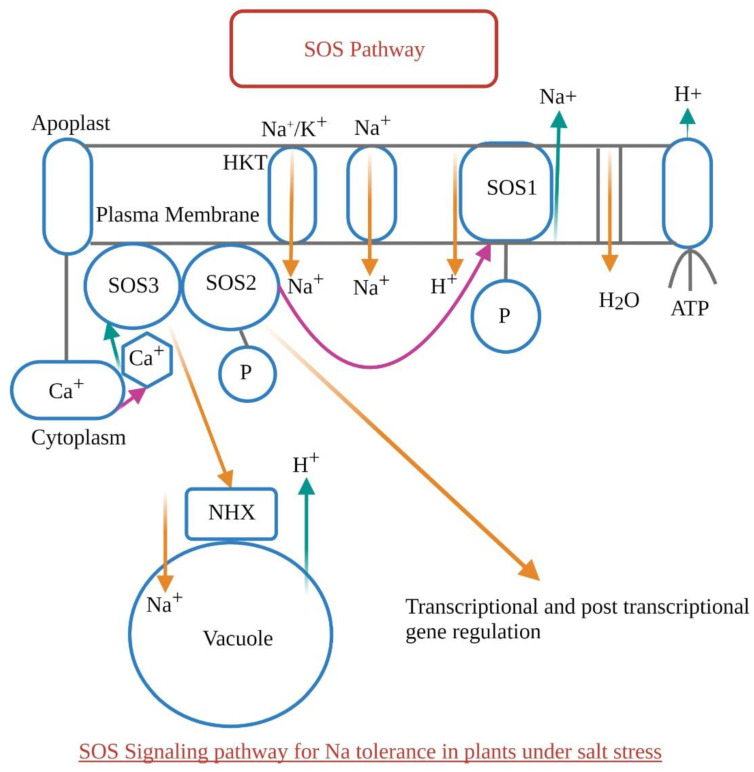
*SOS signaling pathway functions in ion homeostasis and Na^+^ extrusion from cytosol;* A rise in cytosolic calcium is induced by high extracellular salt concentrations. The calcium sensor SOS3 interacts with and activates the protein kinase SOS2 when it detects a signal. The ion transporter activities of transcription factors (TFs) are then regulated by activated SOS2 to regulate ion homeostasis or gene expression. The SOS1 Na^+^/H^+^ antiporter, the NHX vacuolar Na^+^/H^+^ exchangers, and the Na^+^/H^+^ transporter HKT1 are all SOS2 targets. Tonoplast ATPase and pyrophosphates, water channels, and the K^+^ transporter are among the other targets.

**Figure 5:**
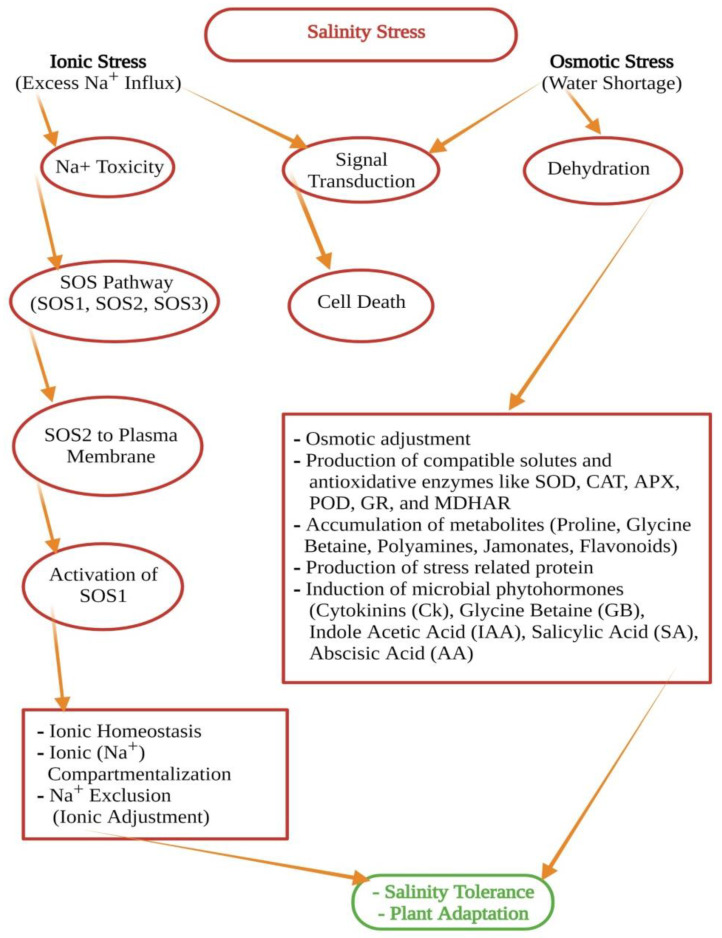
A generalized schematic representation of salt stress in plants leading to ionic and osmotic stress and the resulting tolerance mechanisms including the SOS signaling pathway, ionic homeostasis, osmotic adjustment, the production of metabolites and compatible solutes, rhizosphere microbial activities, and the production of phytohormones to alleviate various stresses, especially salt stress in plants.

The salt stress-induced cellular signaling pathway has three main parts: SOS1, SOS2, and SOS3. These three parts work together to control the concentration of sodium ions in the cytosol. The SOS pathway is a crucial ion homeostasis process in crop plants that transports excess Na^+^ ions for elimination/sequestration. It is an important signaling pathway and a major defense mechanism when plants are exposed to high NaCl concentrations (Figure 4 and Figure 5). However, the mechanism by which components of the SOS pathway are incorporated to facilitate the plant’s tolerance to salinity stress is unclear. The most important genetic mechanism used by plants to resolve sodicity is the SOS pathway [67,68,69]. The molecular and physiological assessments of the SOS pathway have been carried out on glycophytic species using available forward and reverse genetic tools. At the same time, knowledge obtained from halophytes is still limited [26,70,71]. SOS3, a calcium-binding protein, SOS2, a serine/threonine-protein kinase, and SOS1, a Na^+^/H^+^ antiporter, were originally identified as the core components of the SOS Pathway [72].

Ionic homeostasis is regulated by the SOS signaling pathway, a major regulatory mechanism [68]. A calcium signal is triggered by high salinity (Na^+^) stress, which triggers the SOS pathway (Figure 4). In the cell membrane, the calcium-binding proteins SOS3 and SOS2 form a complex that activates the SOS1 antiporter [73]. The purpose of recent research is to isolate, clone, sequence, and characterize the major SOS pathway genes, SOS1, SOS2, and SOS3, from the salinity tolerant plant’s genotype, as well as to investigate the ion accumulation pattern in modified plant clones with varying salinity tolerance [74]. The SOS pathway is dominated by two main processes that lead to salt tolerance. The first one is a cellular process that relies on Na^+^ efflux back to the apoplast or into the soil solution. The control of Na^+^ loading into the xylem, which has been confirmed in Arabidopsis, tomato, and rice plants, is the second, and not least important feature [75]. The role of SOS1 in xylem loading regulation appears to be important for the salt-accumulating halophyte *Salicornia* spp., where the constitutively increased expression of SOS1 in the root may be required to maintain a constant flow of Na^+^ through the xylem to the shoot [76,77].

The involvement of the SOS pathway in salinity tolerance has been explored in different types of plants, i.e., many halophytes, such as *Salicornia brachiata* (sea green bean) [76], *Aeluropus littoralis* (Indian walnut) [78], *Aeluropus lagopoides* (mangrove grass) [79], *Mesembryanthemum crystallinum* (ice plant), *Artiplex gmelini* (saltbush or orache), *Beta vulgaris* (beetroot) [80], *Hordeum brevisubulatum* (wild barley) [81], and *Avicennia germinans* (black mangrove) [82]. C4 plants have a greater potential to manage the photosynthetic apparatus against oxidative stress than C3 plants, making them more immune to salinity stress [83]. C3 and C4 plants, including *Vitis vinifera* (wine grape) [49], *Brassica juncea* (brown mustard) [84,85], *Oryza sativa* (rice) [86], *Triticum aestivum* (wheat) [87], *Medicago tranculata* (barrel clover) [88], and C4 plant species, like *Sorghum bicolor* (great millet) [89], *Zea mays* (corn) [90], and *Saccharum officinarum* (sugarcane) species [91]. Plant salt tolerance is subsequently increased by overexpressed genes in the SOS pathway [92]. One possibility is that SOS2 and SOS3 cannot operate well with SOS1 in shoots, since SOS3 is primarily found in roots. It is also possible that SOS genes interfered with previously unknown factors during plant salt-stress acclimation responses. The optimal co-expression of SOS genes and unknown factors in specific cell types/tissues might help to increase salt tolerance. In plants grown under high salt concentrations, vacuolar partitioning of Na^+^ is a predominant adaptive mechanism for reducing cytoplasmic ion toxicity. This mechanism is conserved in halophytes and glycophytes [93].

### 1.7. Different Channels Involved in Na^+^ Regulation

Different channels are involved in the regulation of salt in plants, i.e., HKT (high-affinity K^+^ channel), NSCC (nonselective cation channel), AKT1 (Arabidopsis K^+^ transporter1), NORC (nonselective outward-rectifying conductance), and VIC (voltage-independent channel). Although a particular transport system for Na^+^ uptake has not been identified yet in plants, Na^+^ inflows across the plasma membrane possibly take place through the high-affinity K^+^ channel (HKT), the weakly voltage-dependent nonselective cation channel (NSCC), the low-affinity K^+^ channel, e.g., Arabidopsis K+ Transporter1 (AKT1), nonselective outward-rectifying conductance (NORC), and/or the voltage-independent channel (VIC). These channels can mediate Na^+^ and K^+^ inflow into plant cells, though only a few have a higher selectivity for K^+^ than for Na^+^ [36].

Out of all these channels, HKT1 has been a key determining factor of plant salinity tolerance in response to salt stress [94]. HKT1 may increase salt tolerance by reducing Na^+^ accumulation in the shoot tissues. It protects leaves from Na^+^ toxicity. Tissue-specific HKT1 manifestations, such as in the pericycle or vascular bundle, improve salt tolerance in the whole plant [95]. Arabidopsis HKT1 is intensely articulated in the root stelar cells and leaf vascular tissue (Figure 4). HKT1 mutants are sensitive to salt stress and accumulate more Na^+^ in shoots than in roots, indicating that HKT1 aids in the distribution of Na^+^ between roots and shoots by relocating Na^+^ from the root to the shoot xylem [96].

In both the SOS2 and SOS3 mutants, HKT1 mutations suppress the salt-hypersensitive phenotypes, suggesting that HKT1 coordinates with the SOS (salt overly sensitive signaling) pathway to modulate Na^+^/K^+^ homeostasis in plant cells [97]. Many HKT loci have now been cloned in different plant species due to genome-wide association studies (GWAS)—a genetic approach to associate natural genetic variations with specific traits— and quantitative trait locus (QTL) analysis to assess genetic variations in plant salt tolerance. This demonstrates that HKT proteins have been selected for plant salt resistance during evolution and in plant breeding programs [98,99]. Plants use HKT proteins as a crucial line of defense against high salinity, so there is considerable natural variability that can be used in captive breeding [100,101]. As a result, we will spend the remainder of this analysis updating our understanding of HKT protein structure and function, as well as their role in plant genetic improvement and conservation efforts.

### 1.8. Metabolic Profiling; Osmolyte Production in Plants during Salt Stress

Different types of metabolites and their dynamic responses to various stresses has been observed in variety of plants. As secondary metabolites are produced and activated in different plant processes, they become more essential [29,102,103]. Sugar beet plants produce dimethyl compounds, which seem to be effective osmoprotectants and thus good antioxidants, enhancing plant resilience to salt stress. A few of these compounds often help plants boost their energy capacity by increasing photorespiration during stressful conditions [22]. Crop plants must deal with oxidative and ionic stress; both of these conditions arises in plants in salty environments. According to certain studies, salt tolerance may also boost the plant’s resistance to oxidative and water stress.

To maintain turgor pressure under chronic salt stress, salt-tolerant plants deposit adequate solutes in their cytoplasm. Despite numerous research efforts to better understand the processes that plants adopt to cope with or avoid salt stress, the processes of salinity tolerance in plants, particularly at the genome level, are still largely unknown. Salt stress, on the other hand, has been linked to the expression of a wide spectrum of metabolites and their modifications in several studies [104,105]. The biosynthesis and aggregation of compatible osmolytes are induced by activating salt-mediated osmotic stress pathways (both short- and long-term) to reduce the cell osmotic potential and regulate proteins and cellular structures; this is a key adaptive technique [106]. Salt stress has been shown, in many studies, to cause the expression of various complex metabolites and their modifications. This study examines representative categories of metabolites, their genetic foundation, and the genes that control these metabolites. These metabolites aid plant growth to thrive in and combat salty environments.

In response to salt stress, plants accumulate metabolites (Table 1) such as proline and its derivatives (proline betaine, glycine betaine, etc.), sulphonium compounds such as choline-O-sulfate, dimethylsulfoniopropionate, the raffinose chain of sugars, phenolics, glycerides, mannitol, sorbitol, galactose, trehalose, fructose and dimethyl inositols [107,108,109]. However, it is beyond the reach of this review article to include all these metabolites. As a result, we will only discuss glycine betaine (GB), proline, abscisic acid (ABA), jasmonates, and flavonoids, which are among the most important metabolites. Some of the metabolites, such as proline, glycine betaine, polyamine, mannitol, glucose, fructose, and trehalose, are typically accumulated in several plant species in both the roots and the leaves in response to salt, heat, drought, ionic, osmotic, and heavy metal stresses, whereas sugar is primarily accumulated in shoot tissue. However, some of these, such as choline-O-sulfate, b-alanine betaine, and hydroxy-oxyproline, are likely to be accumulated in specific species.

Secondary metabolites (phenolic compounds) are plant antioxidants that are generated in response to stress. Tocopherol, which aids in membrane integrity [117,118], and ascorbic acid, carotenoids, flavonoids, and glutathione are examples of secondary metabolites. Dimethylsulfonium propionate, glucosyl glycerol, and glycerol are some of the metabolites that are primarily accumulated in marine algae and *Dunaliella parva*. These metabolites can directly bind to and activate or inactivate enzymes to control salt reactions in addition to their osmotic and detoxification functions. However, further research into these practices is needed [119].

### 1.9. Salinity Stress and Proline (A Crucial and Multifunctional Amino Acid That Can Affect Plant Growth as Well as Stress Responses)

All crop plants contain proline, which increases as the plants are subjected to various stressors. In response to biotic stresses, extreme salinity, intense light, toxic substances, and cellular antioxidant activities, proline aggregation has been observed. [120]. Proline is a reliable and practical criterion for determining the crops ability in tolerating stress, such as salinity. Proline levels will rise because of salt stress [121]. It is also essential for the osmotic modification and stabilization of various enzymatic and proteomic activities. Furthermore, under abiotic stress, free proline increases in plant tissues, which aids in scavenging unwanted free radicals. Proline is thought to scavenge ROS indirectly by increasing antioxidant enzyme activity rather than specifically. It is said to be more effective than glycine betaine at reducing the adverse effects of salinity [122]. Higher concentrations of proline can be lethal to plants, causing ultrastructural disruption and generating reactive oxygen species (ROS) [123]. Stress causes a rapid breakdown of proline, resulting in ATP production as an energy supply to recover and repair stress-induced damage [124]. Proline accumulates in the cell cytosol during salt distress and plays an important role in osmotic modification [125,126].

In Arabidopsis, research has revealed an improvement in cytosolic proline [127]. When different plant species were exposed to salt stress, their proline concentration increased, and as a result, these plants exhibited increased stress tolerance [128,129,130]. Under salt stress, for example, elevated proline concentrations were observed in *Medicago sativa* (alfalfa/lucerne) and *Medicago truncatula* (Alfalfa spp./barrel medic) relative to control plants [131]. These findings suggest that proline formation in agricultural plants in response to environmental stresses is well understood, while there is little knowledge on the signaling mechanism that regulates production of Ca^+^ [132], and abscisic acid appears to be implicated in proline biosynthesis. Proline accumulation was previously reported in plants under stress, where it has emerged as a salt stress injury symptom, for example, in rice [133] and sorghum [130]. In salt-stressed potato seedlings, the pyrroline-5-carboxylate synthetase (P5CS) activity increased, while the activity of proline dehydrogenase decreased. In salt-sensitive cultivars, these improvements in enzymatic expression were more pronounced [121,134]. However, salt exposure resulted in an increase in proline content in potato clones [135].

A transgenic method for improving plant salt-stress resistance has had some positive results in past years. Tobacco plants transformed by the P5CS gene reported enhanced proline concentration and were found to be immune to salinity stress [136]. Increased salt-resistance and a rise in antisense proline dehydrogenase were observed in cDNA-engineered Arabidopsis plants [137]. Ethephon raised proline levels in spinach when paired with sodium chloride [138]. The presence of proline in bacteria linked to plants underwater or salinity stress emphasizes the role of proline. Plants can use high proline levels as a nitrogen supply during regeneration [139]. Exogenous proline, for example, has been shown to impart salinity tolerance in rice by regulation of its antioxidant defense mechanisms [140]. The effective amount of proline depends on the genotype and stage of growth of the plant [141,142]. Drought-prone and drought-resistant barley genotypes cultivated in saline environments were shown to accumulate proline. A considerable amount of proline was present under salt stress, with small quantities in root tissues. Proline deposition is presumed to be more predominant in the receptive genotype [143]. Under stressful conditions, there is a greater concentration of proline in the cell cytosol, strengthening the cell’s capacity to make ionic changes. Its accumulation is proportional to plant stress resistance [144]. Exogenous proline application may be another choice for crop plants to increase their resistance to salt stress. So far, proline deposition has responded differently to various plants at different salinity levels.

### 1.10. Salinity Stress and Glycine Betaine (An Effective Protectant against Abiotic Stresses in Plants)

From bacteria to higher plants and mammals, glycine-betaine (GB) is found in various species when they are exposed to different environmental stresses. It preserves and regulates the performance of PSII (photosystem II) protein complexes by shielding extrinsic regulatory proteins from denaturation, as well as osmoregulation. It also stabilizes macromolecules by forming close bonds with water [145]. It is often referred to as an “osmo-protectant” because it prevents these macromolecules during drought and heat stress [146]. When GB was sprayed onto rice plants under salt stress, it boosted salt resistance considerably. [147]. When applied to tomato plants, it resulted in a forty percent on average increase in fruit yield when compared to control plants. [148]. In comparison to untreated plants, GB-treated plants had slightly lower Na^+^ and higher K^+^ ions in their shoots. GB maximizes plant resistance to salt stress by increasing the retention of Na+ in the roots and decreasing its transit to the shoots by forming more vacuoles in the root cells. SOS is a unique transduction channel that is predominantly controlled by MAP kinases, and GB was shown to significantly influence the expression of these enzymes [68]. These findings indicate that GB’s signal transduction and ion homeostasis function can significantly influence salt tolerance. The most important source of GB is the sugar beet [149]. Regarding pure GB, it is valued as a desirable source of GB and other beneficial compounds. GB is effective in promoting vulnerability to salt stress in eggplants (*Solanum melongena* L.). It has a massive effect on morphological (growth and yield), physiological, and biochemical (gas exchange, photosynthetic) processes [150].

This research indicates that GB’s influence on signal transduction and ion homeostasis can play a role in salt tolerance. Genetically modified crops possessing GB-synthesizing genes, such as mustard, rice, Arabidopsis, and tobacco, have increased GB production and improved tolerance to a variety of stressors, including salinity [148,151,152]. However, the increase in GB in genetically engineered crops is only a fraction of what occurs naturally when plant species are under stress. This is owing to a lack of choline availability or limited choline transport into the chloroplast. As a result, genetically modified (GM) crops are also limited to producing GB to the alleviate salt strain. As a result, other concomitant factors, such as substrate choline and metabolic flux supply, should be considered when engineering crop plants for GB over-production.

Choline is the precursor for GB, and enzymes including choline monooxygenase and betaine-aldehyde dehydrogenase control the conversion [153,154]. Choline supplementation of the salt-stressed plant’s growth media will help to regenerate the plant’s stunted growth [155]. Other concurrent parameters, such as the availability of the precursor choline and biochemical fluxes, should be explored in breeding crop plants for excessive GB production. Endogenous GB treatment could be a viable option for increasing salt stress resistance in crop plants. In contrast, studies have shown that such applications can result in significant growth and maximum production in agricultural plants, such as tobacco crops and soybean. [156]. Exogenous application of GB has been observed to improve the productivity, leaf water quality, and net photosynthesis of salt-stressed pea plants [157]. Improvements in stomatal conductance and PSII efficiency are assumed to be involved in the increase in net photosynthesis in maize plants. Furthermore, for a successful treatment, a greater knowledge of the pathways of externally applied GB is required.

Glycine-betaine accumulates in some stressed crop plants, such as the family of Poaceae (grass family) and Chenopodiaceae (flowering plant family), but is absent from others, such as rice and tobacco. Therefore, scientists have sought to create transgenic plants that can produce GB. If transgenic plants accumulate GB, their reproductive organs can withstand abiotic stresses [158]. GB is a water-soluble compound that accumulates primarily in plastids and chloroplasts and is not toxic at higher concentrations. Exogenous GB stimulates salinity tolerance in plant species that do not generate it naturally. Plants can absorb exogenously introduced GB via their leaves [159] and roots [160]. Following absorption, GB is translocated into the phloem. GB isn’t active in scavenging ROS plants in any way. It reduces the harmful effects of reactive oxygen species (ROS) by encouraging enzymes that destroy or inhibit ROS activity [161]. The reproductive organs of plants accumulate more GB than the vegetative portions in order to avoid stress conditions [142,162].

Sugar beets, spinach, rye, barley, and sorghum are among the natural GB-accumulating species. Increased resistance is directly proportional to GB conception. Increased resistance to abiotic stresses, especially salt stress, is mainly due to osmotic adjustment. GB oversees maintaining turgor via osmotic adaptation [163,164]. This partnership, though, is unsatisfactory in some Triticum and Agropyron species. [165]. By sustaining a high K+ concentration in contrast to Na+ ions, GB assists in osmotic tolerance and ionic homeostasis. The K+/Na+ ratio was boosted when GB was applied exogenously [166,167,168]; GB also controls the photosynthetic apparatus. It stimulates photosynthetic activity by reducing photorespiration and increasing stomatal conductance [169,170,171].

Some studies have suggested that exogenously induced GB has neutral or slightly harmful effects on some plant genotypes. The rate, length, timing, and frequency should all be considered when using GB commercially. By deep study and comprehensive reading analysis, we can conclude that exogenously implemented GB enhances salt resistance in rice plants by increasing antioxidant levels and enhancing relative water content in the leaves. Lipid peroxidation is seen to be reduced by GB. The primary source of GB has been identified as sugar beets. GB is more effective than proline at preventing membrane adulterations caused by osmotic stress.

### 1.11. Salinity Stress and Abscisic Acid (A Ubiquitous Plant-Stress Hormone)

Abscisic Acid (ABA) is an isoprenoid plant hormone that occurs naturally. When plants are subjected to salt stress, the concentration of ABA rises proportionally. When plants are exposed to salt stress, their Ca^2+^ absorption increases, and their ABA levels rise. This greater level of abscisic acid helps plants retain structural fluidity and regulate, absorb, and transport ions more effectively [172]. At the transcriptional level, ABA is accountable for the upregulation and downregulation of salt-related genes in morning glory flowers, Arabidopsis thaliana Na+/H+ exchanger (AtNHX1), and sweet potato, resulting in the aggregation of Na^+^ in the vacuole [173]. Agricultural crops have been demonstrated to be protected from the cytotoxic activity of salt stress on a variety of systems, including photosynthesis, due to the presence of ABA.

ABA has been found to mediate signaling in plant cells under various environmental pressures. To generate compatible osmolytes, stress-related genes are expressed. Osmotic stress causes plants to accumulate ABA, which modulates the expression of several genes. [174]. The quantities of indole-3-butyric acid (IBA) in developing foliage are significantly higher in salt-resistant maize. Calcium is involved in the ABA-mediated activation of the pyrroline-5-carboxylate synthase (P5CS) gene during salt distress. These hormonal changes can help to provide optimal conditions for growth-promoting substances, such as cell wall expansions. Increased ABA levels can lead to apoplast acidification [175].

### 1.12. Jasmonates (Lipid-Derived Plant Stress Hormones)

Jasmonates are cellular regulators that play a role in a variety of plant developmental processes. Plant defense mechanisms against pathogens and environmental threats, such as salt stress, are activated by jasmonates. Biosynthesis begins with wounding caused by various methods, or by pathogens attacking cells that generate fatty acids by disrupting cell membranes [176]. Allene oxide synthase and lipoxygenases (LOXs) are enzymes involved in the synthesis of jasmonic acid. In response to abiotic stress, jasmonates trigger the synthesis of many proteins. These proteins help plants defend themselves against various stresses, including salt stress [177]. Under salt-stress conditions, a salt-resistant cultivar had a higher JA amount (enhanced JIPs) than a salt-sensitive cultivar [178].

All of this research suggests that jasmonates are essential metabolites that play a role in plant salt tolerance. Exogenous application of nutrients and metabolites to plants is a popular strategy for various nutrients and metabolites. The exogenous application of JA will help salt-stressed rice seedlings recover [179], and sodium levels drop drastically. The effects of salinity on the endogenous JA level in plants, on the other hand, are little understood. To completely understand the involvement of JA in salt stress, more research is needed, particularly at the molecular level. This could pave the way for the development of novel JA compounds or derivatives that directly disrupt the metabolic mechanisms that induce stress in plants cultivated in saline environments. Furthermore, when plants are under salt stress, this could lead to the synthesis of potential chemicals that boost stress-releasing activities.

### 1.13. Polyphenols/Flavonoids (Important Secondary Metabolites and Bio-Compounds in Plants)

Flavonoids are secondary plant metabolites with over ten thousand structural variants and a wide range of activities. Higher plants that are subjected to a variety of environmental pressures have an antioxidant role. Because flavonoids are good ROS scavengers, plants acquire them during times of stress [180]. During various stressful situations, reactive oxygen species (ROS) production is a natural phenomenon. Consequently, when plants are stressed, such as by salt, the biosynthesis of antioxidant flavonoids rises. The role of flavonoids in saline conditions has been discovered in sea grass (*Halophila johnsonii*), which contains 15 different polyphenols, 10 flavonoid glycosides, and 5 flavanols. [181]. It has been reported that flavonoid amount contributes more strongly to salinity variation than genetic control because plants differ in phenolic contents and localization in response to salinity variation [182]. Flavonoids are found in the cuticle and cytosol of both abaxial and adaxial leaf epidermal cells in intertidal seagrass [183].

However, it is still unknown whether flavonoids act as antioxidants when detected in the cytoplasm of epidermal leaf cells. In such situations, greater energy requirements for development may signify increased metabolic rate to maintain intracellular electrochemical equilibrium, resulting in leaf area reductions. The chloroplast and other cellular organelles produce reactive oxygen species (ROS), which flavonoids may scavenge. Activated antioxidant metabolism is indicated by increased Trolox equivalent antioxidant capacity (TEAC) values in saline conditions. This suggests that flavonoids play a key role in salt tension, and further research into the topic is needed.

Moreover, rhizospheric microbes can also be utilized to improve plant systemic tolerance to biotic and environmental stress. *Bacillus subtilis*, through a biocontrol mechanism, kills microorganisms that cause disease either directly or indirectly [184]. Plant development is enhanced by the synthesis of water-soluble vitamins, such as niacin, by some *Pseudomonas* species. Furthermore, particular mycorrhizal fungi that act as plant symbionts, such as *Trichoderma harzianum*, can be extensively utilized to increase plant tolerance to abiotic or biotic conditions, such as drought and salinity. Biofertilizers are recommended as environmentally friendly options, since they aid in phosphorus solvation, nitrification, ammonia formation, enzyme function, and the production of a variety of plant hormones. They also have biocontrol properties against a wide range of plant-pathogenic agents. The colonization of bacteria and fungus into the soil or seedling roots can populate the rhizosphere or the interior parts of the plants, promoting plant growth and development [185].

## 2. Conclusions and Future Perspectives

Plants must be able to change their development to adapt to stressful situations. One of the most important abiotic pressures that plants encounter is salt stress. Identifying and characterizing upstream salt stress sensors could help lead to strategies for reducing the detrimental impacts of salt stress on crop yields and, in turn, improving agricultural growth. Plants have evolved regulatory mechanisms that allow them to adapt to these harsh conditions, but salt stress has a negative impact on their growth and development. Salt stress, for example, inhibits plant growth by reducing photosynthesis. Plant cells undergo significant modifications to respond to and protect against salt stress [186]. The plant loses water, which damages the membranes, and the photosynthetic pigment concentration is likewise lowered as salinity rises. On the other hand, exogenous calcium ions enhance the degree of membrane peroxidation disrupted by high salinity, boost plant photosynthetic capacity, and reduce cytotoxicity caused by the fast increase in Na^+^. The addition of Ca^2+^ causes the steady-state of Na^+^ and K^+^ to be balanced, which is the most fundamental factor in reducing salt stress [114].

Salt-stress-related gene screening requires high throughput and efficient biotechnologies. RNA sequencing has been shown to be a quick and successful tool for understanding the molecular regulation of salt tolerance in plants. Novel genes related to the regulation of the plant salt stress response have been identified using transcriptome sequencing approaches [187,188]. The advancement of next-generation sequencing technology has made finding salt-tolerance genes much easier [189]. The use of RNA-Seq to conduct a global study of plant transcriptome profiles and microRNA levels in response to salt stress yielded useful insights into salt tolerance mechanisms [56]. These discoveries also provide a valuable resource for integrating salt-related genes into biotechnological approaches to develop salt-tolerant crops.

Over the last few decades, significant progress has been made in understanding the genetics and physiology of salinity tolerance in plants. Several genes have been discovered that confer salt tolerance. As a result, forward genetics will be used to identify a huge number of genes. Molecular responses to salt stress are still poorly understood, from sensing and signaling to the creation of adaptive tolerance mechanisms, and further research is needed. To clearly separate the osmotic and Na^+^ stress responses in plants, it is critical to identify upstream pathways and molecular processes involved in salt stress sensing [190]. Using bioinformatics approaches, we can identify genes related to salt stress in order to determine the phenotypic and genotypic diversity of numerous plant cultivars grown in various climatic zones. As a result, more research is needed to develop breeding techniques under abiotic stress and stress response mechanisms and signaling networks [19]. Advanced approaches, such as transcriptomic mapping, could be utilized to decipher metabolic changes at the gene expression level. Metabolomics profiling, on the other hand, is commonly utilized for different stressors in diverse plant species, such as cannabis and cereals. Metabolomics effectively elucidate stress-related pathways or targets for improving crop resistance [136,190].

This review covers what happens to plants when they are exposed to salt stress and the physiological response that follows, which primarily involves osmotic adjustment and ROS scavenging, and includes four main signaling pathways, the corresponding salt stress-responsive genes, and some plant salt tolerance improvement techniques. Over the last two decades, significant progress has been made in understanding the mechanism of plant salt tolerance. Nevertheless, there is still a lot to discover and explore in the future. To begin with, the mechanism by which salt stress reduces plant photosynthesis is unknown. So far, no unified understanding has emerged. Second, future research is needed to integrate morphological, physiological, and molecular techniques for plant stress detection. Third, there is still much to learn about halophytes’ particular salt-tolerant mechanisms, such as salt vesicles. Fourth, the function of salt stress is unknown, and the cross-reactions of multiple signal transduction pathways in salt-stressed plants are poorly understood.

In the future, comparative genomic and transcriptome approaches may be used to discover more salt stress-responsive genes. Finally, we must determine how to prioritize the importance of each improvement technique to attain better results. Overall, we still have a long way to go in understanding the mechanism of plant salt tolerance and its enhancement [191]. Future studies should focus on creating high-density mapping populations by crossing salt-resistant and salt-sensitive varieties; these species can then be used for high-throughput phylogenetic analysis and sequential cloning [119]. In summary, although we have learned a great deal about plant salt-tolerance mechanisms in the last few years, there is still much more that we do not know.

## Figures and Tables

**Table 1:** Osmolyte production in plants in response to salt, drought, heat, and osmotic stress.

Type of Abiotic Stress	Secondary Metabolites/Osmolytes Production	References
Salt stress	Proline, Glycine Betaine (GB), Flavonoids, Jasmonates (JA), Abscisic acid (ABA)	[110,111,112,113,114]
Drought stress	Proline, Glycine Betaine (GB), Polyamines	[3]
Heat stress	Abscisic acid (ABA), Glycine Betaine (GB), Proline, Polyols	[111,113,115]
Osmotic stress	Glycine Betaine (GB), Polyamines	[57,58,116]

## Data Availability

The data presented in this study are available in the figures and tables.
